# A Comprehensive Review on Hot Ambient Temperature and its Impacts on Adverse Pregnancy Outcomes

**DOI:** 10.34763/jmotherandchild.20232701.d-22-00051

**Published:** 2023-06-27

**Authors:** Shanmugam Rekha, Sirala Jagadeesh Nalini, Srinivasan Bhuvana, S. Kanmani, Venugopal Vidhya

**Affiliations:** Department of Environmental Health Engineering, Sri Ramachandra Institute of Higher Education and Research, Chennai, Tamil Nadu, India; Faculty of Nursing, Sri Ramachandra Institute of Higher Education and Research, Chennai, Tamil Nadu, India; Department of Obstetrics and Gynecology, Sri Ramachandra Medical Centre, Chennai, Tamil Nadu, India; Centre for Environmental Studies, College of Engineering Guindy, Anna University, Chennai, Tamil Nadu, India

**Keywords:** Heat stress, Physical exertion, Physiological strain, Pregnancy outcomes, Low birthweight, Spontaneous abortion, Recommendation

## Abstract

**Introduction:**

High workplace/ambient temperatures have been associated with Adverse Pregnancy Outcomes (APO). Millions of women working in developing nations suffer due to the rising temperatures caused by climate change. There are few pieces of research linking occupational heat stress to APO, and fresh evidence is required.

**Methodology:**

We used databases including PubMed, Google Scholar, and Science Direct to search for research on high ambient/workplace temperatures and their effects. Original articles, newsletters, and book chapters were examined. The literature we analysed was categorised as follows: Heat, strain, and physical activity harming both mother and fetus. After categorising the literature, it was examined to identify the major results.

**Results:**

We found a definite association between heat stress and APOs such as miscarriages, premature birth, stillbirth, low birthweight, and congenital abnormalities in 23 research articles. Our work provides important information for future research into the biological mechanisms that create APOs and various prevention measures.

**Conclusion:**

Our data suggest that temperature has long-term and short-term effects on maternal and fetal health. Though small in number, this study stressed the need for bigger cohort studies in tropical developing countries to create evidence for coordinated policies to safeguard pregnant women.

## Introduction

Over the next several decades, global climate change is anticipated to increase the global temperature by at least 1.5°C [1]. India is a tropical nation with sweltering summers in various locations that could potentially jeopardise the health of a few million people [2]. Workers, especially those exerting themselves outdoors, are affected the most as during India's warmest season, when the daily climate is unsuited for manual labour [3]. Heat stress, which is caused by a combination of ambient temperature, humidity, sun radiation, and wind speed, can cause adverse physiological responses if it lasts for an extended period of time [4]. The effects of heat stress can range from mild discomfort to death [5].

According to the 2012 Census, India's total population is 1.22 billion people, including 591.4 million women [6]. The informal sector (those with no fixed employment terms; these sectors are not registered with the government), which employs about 60% of women [7], many of them of reproductive age, is home-based for activities that require prolonged exposure to heat and severe physical labour. With the rise in female participation in strengthening the economy, many women are expected to work during their reproductive years and even after giving birth, highlighting the significance of empowering them with health and independence. This increases the risk of pregnant women being exposed to occupational heat stress, which can result in Adverse Pregnancy Outcomes (APOs) like spontaneous abortion, stillbirth, premature birth, preterm delivery, a small gestational age, low birthweight, and congenital defects [8,9,10,11]. Women continue to work during heat waves, and during heat waves [12], a pregnant woman's ability to control her body temperature is extremely sensitive [13], which can create difficulties at any stage of the pregnancy [14].

These factors have a significant impact on maternal and fetal outcomes [15–16]. As the baby's temperature is one degree higher than the mother's, it may be unable to cool down in the uterus through sweating, resulting in heat stress, which has been linked to birth defects like congenital heart diseases and abdominal wall defects [17]. Excessive dehydration and a lack of fluid replenishment can put a woman's health in jeopardy and make her more vulnerable to the negative effects of high-heat loads, which can cause a decrease in blood volume and an increase in blood concentration, which increases the amount of available oxytocin, the hormone that causes contractions that can cause early labour and post-natal complications [18]. Because organogenesis begins during the first trimester of pregnancy, excessive heat stress may cause congenital cardiac and neurological abnormalities [19].

Following the achievement of the Millennium Development Goals, the World Health Organization has made enhancing maternal and child health a priority in its Sustainable Development Goals [20]. APOs play a significant role in global morbidity and mortality, affecting expectant mothers and their children throughout their lives [21], and preterm delivery affects 10% of all pregnancies worldwide [22]. To prevent unfavourable health consequences, more research is needed to identify vulnerable populations, fill knowledge gaps, and implement strategies for heat prevention and mitigation.

This systematic review aims to compile all known evidence-based research on the effects of heat stress on APOs. It is crucial to demonstrate the association between high temperatures and unfavourable maternal and fetal outcomes, so we analysed the current literature on heat stress to determine its consequences. Our findings will aid in defining the problem's magnitude, determining research priorities, control strategies, and presenting scientific evidence for policy reforms where applicable.

## Methodology

**Figure 1.**
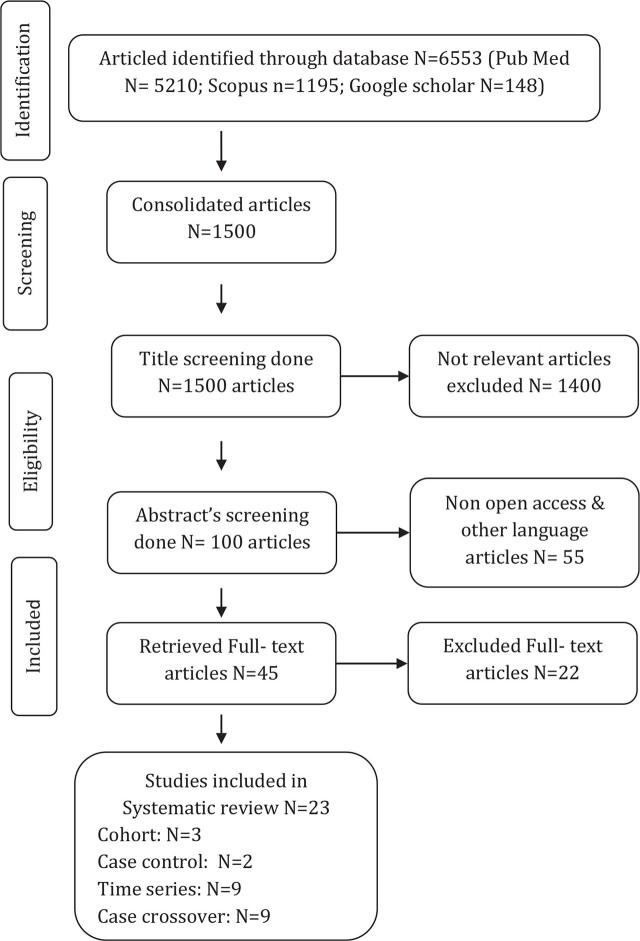
PRISMA flow diagram of the study selection process.

To examine the impact of heat stress on maternal, fetal, and neonatal health, we conducted a literature survey (Figure 1). We also searched for relevant research publications published up to December 2021 in databases such as PubMed, Scopus, and Google Scholar. Case control studies, cross-sectional studies, time series studies, and longitudinal studies were all evaluated. Commentaries, editorials, and letters to the editor were not included in the publication. We restricted our search to English, and all relevant articles’ reference lists were checked for any additional relevant research.

### Data Extraction

To find relevant articles, we utilised the following search criteria: AND pregnancy AND human beings AND temperature (OR ambient temperature OR high temperature OR hot temperature OR heat wave OR climatic change) (OR pregnancy outcome OR spontaneous abortions OR miscarriage OR gestational age OR Stillbirth OR birth weight OR preterm birth). This review examined papers on reproductive health, maternal health, birth outcomes, working women, and extreme heat. The impact of high ambient temperatures and heat waves on maternal and fetal health is the subject of this study. We eliminated research papers that did not include human population studies or dealt with topics unrelated to heat stress. The research articles were evaluated using the following criteria: exposure population, study type, location, type of exposure, measurable result, and potential sources of bias. For our review, we looked at the following definitions: The termination of a pregnancy before the 20^th^ week of pregnancy or the delivery of a baby weighing less than 500 grams is considered an abortion [23,24,25]. When a baby is born before the 37^th^ week of pregnancy, it is known as preterm birth (PTB) [22]. The World Health Organization (WHO) defines low birthweight (LBW) as a birth weight of less than 2500 grams at term (5.5 lb) and stillbirth as the birth of a baby who shows no signs of life at or after 28 weeks of pregnancy [26–27]. Following that, data extraction for the review was completed.

### Data synthesis

To determine exposure, we obtained data on temperature fluctuations and the length of heat episodes. Studies were included and examined for eligibility if they met the PICO components: population, intervention, comparator, and outcomes. We treated pregnant women and newborns as the target population; exposure to high temperatures as the intervention; hot and cold seasons as the comparator; and unfavourable maternal and fetal outcomes such as miscarriages, PTB, LBW, and stillbirths as the outcomes.

For each category of health outcome, we acquired statistical data, risk estimates, and confidence levels where available. Confounding data was also obtained, such as socioeconomic characteristics, diet, and air pollution. Each article was evaluated for biases such as selection, misclassification, detection, and internal validity. The Preferred Reporting Items for Systematic Reviews and Meta-Analyses (PRISMA) criteria were used to select studies [28]. Only 23 publications were found to match the review's final selection criteria, out of a total of 1500. Endnote was used to export all of the search results. The paper's title, abstract, and full text content were all examined first. We found both review papers and original research publications as a result of our search; however, we did not include review articles in our analysis. As a result, the final selection of articles was determined based on the search keywords, the reference lists of the retrieved articles for cross-referencing, and related citations from the published literature.

## Results and Discussion

Our comprehensive literature review revealed that warmer ambient temperatures have a greater potential impact on APOs. In the majority of the analyses, the relationships between rising temperatures and APOs appear to be greater and more persistent. Approximately 23 studies have discovered an association between heat exposure and unfavourable pregnancy and delivery outcomes (Table 1). However, we discovered just three studies that examined the impact of heat stress on pregnant women.

**Table 1:** Summary of study characteristics, significance within individual study findings

**S.No.**	**Author, Year**	**Location**	**Study Design**	**Causal factor**	**Maternal outcome**	**Fetal outcome**	**Exposure window**	**Concluding remarks**
**Studies conducted among non-working pregnant women**								

1.	Sun et al., 2019 [83]	United States (403 countries)	Retrospective cohort study	Extreme heat	-	PTB	35 Weeks	Higher number of heat days associated with higher risk of PTB.
2.	Shashar, et al., 2020 [48]	Unites States	Cohort study	Temperature	Preeclampsia	-	1 or 3 rd trimester	Warm seasons are associated with an increased risk of preeclampsia.
3.	Van Zutphen et al., 2012 [38]	United States	case–control study	Heat exposure	Multiple birth	LBW, PTB	Weeks 4–7	Multiple ambient heat exposure indicates occurrence of LBW, PTB & congenital cataracts.
4.	Zhang et al., 2019 [64]	United States	case–control study	Heat exposure	-	Fetal malformations, Congenital heart diseases	3–8 weeks post conception	Summer may see an increase in the burden of Congenital Heart Disease (CHD).
5.	Auger et al. 2014 [8]	Montreal, Canada	Retrospective cohort study	Hot ambient temperature	-	Early birth	1 week prior to delivery	Exposure to very high ambient temperatures may decrease the gestational duration of warmer months pregnancies.
6.	Basu et al. 2010 [84]	California, USA	Case crossover	Ambient temperature	Chronic infections and hypertension	PTB	1 week prior to delivery	The apparent temperature and premature delivery rates are higher during the warm season.
7.	Kent et al. 2014 [85]	Alabama, USA	Case crossover	Heat wave	-	PTB	1 week prior to delivery	Heat wave days were associated with PTB.
8.	Kloog et al. 2015 [86]	Massachusetts, USA	Time series	Air Temperature	-	PTB, LBW	Full gestation	Increases in ambient temperature were linked with a reduction in gestational age.
9.	Kloog et al. 2018 [74]	Southern Israel	Time series	Temperature	-	PTB	Full gestation	Temperatures as predicted by predictive modelling and risk is high (PTB)
10.	Basu et al., 2017 [87]	Northern California, USA	Case crossover	increased heat percent change per 10°F (5.6°C) increase in apparent temperature	-	PTB	Full gestation	Warm season has a higher risk than the cold season.
11.	Basu et al., 2018 [88]	California, USA	Time series	Extreme temperature	-	LBW	First and third trimester	All results are shown as a percentage change in the OR of LBW with a temperature rise of 10°F.
12.	He et al. 2016 [89]	Guangzhou, China	Time series	Ambient Temperature	-	PTB	Full gestation	Pregnancy-related exposure to both low and high temperatures was linked with an increased risk of PTB.
13.	Dadvandet al. 2011 [90]	Barcelona, Spain	Time series	Extreme heat	-	Early birth	1 week prior to delivery	Average gestational age of birth reduces after maternal exposure to severe HI episodes.
14.	Dadvand et al. 2014 [75]	Barcelona, Spain	Time series	heat exposures	-	LBW	Full gestation	Increased risk of term LBW associated with heat exposure.
15.	Vicedo-Cabrera et al. 2015 [91]	Stockholm, Sweden	Time series	Heat	-	PTB	4 weeks prior to delivery	Exposure to moderate heat during the last month of pregnancy increases the chance of PTB.
16.	Strand et al. 2012 [9]	Brisbane, Australia	Time series	High temperatures	Maternal hyperthermia	Early birth, PTB, still birth, abortion	4 weeks prior to delivery	The risk of stillbirth was similarly increased when the past four weeks of temperature exposure were used instead of the most recent week.
17.	Mathew et al. 2017 [92]	Alice Springs, Australia, Brandenburg and Saxony	Time series	Ambient Temperature	-	PTB	3 weeks prior to delivery	Temperature values have an impact on the consequences and risks of preterm birth at both the lowest and maximum temperatures.
18.	Asamoah et al., 2018 [30]	Multi-country representative survey	A cross-sectional study	High ambient temperature	Miscarriage	Still birth, Congenital abnormalities	Yearly average and monthly average for second month of pregnancy	Environmental heat exposures may be associated with APOs.
19.	Lyndsay A. Avalos [93]	Northern California	case-crossover study	Warmer season	-	Pre-Term birth	Full gestation	Evidence for an increase in the odds of spontaneous PTD associated with increases in apparent temperature.
20.	Jeroen de Bont et al., 2022 [94]	Sweden	Case-crossover,	Ambient temperature d	-	preterm birth	Full gestation	Higher ambient temperature demonstrated increased risk of extremely preterm birth

**Studies conducted among working pregnant women**								

21.	Flocks et al., 2013 [95]	Hispanic and Haitian nursery	Cross sectional study	Heat	Dizziness, Pre-Existing high and low blood pressure, Nausea, Vomiting, sun stroke, Feverish, Dehydration	Fetus become agitated, increased fetal heart rates, Increased fetal movement	-	Heat exposure can adversely affect pregnancy and fetal health.
22.	Rahman et al., 2016 [10]	Bangladesh	Cross sectional study	Heat stress	Increase body temperature	Fetal destruction or Anomaly	-	Outdoor work during pregnancy in hot, increasing body temperature up to levels that could induce fetal destruction or anomaly.
23.	Banerjee, 2009 [37]	-	Continuing Medical education (CME)	Heat stress	Dehydration	-	-	Women in various occupations have been found to be at an increased risk of experiencing a fetal death.

The physiological responses of women to high temperatures differ from those of men, making women naturally more susceptible to heat risk. They perspire less, have a higher metabolic rate, and have thicker subcutaneous fat, all of which contribute to a reduction in radiative cooling [29]. Radiation exposure can promote heat loss and convection from the body's surface, which may exacerbate pregnancy complications in women who are naturally warm, especially during pregnancy [30].

### Heat stress and physical exertion

Pregnant women can work throughout their pregnancy for a variety of reasons, including financial necessity, insurance preservation, postpartum leave time, and job security [31]. According to Palmer et al. [32], workplace characteristics such as work hours, shift work, lifting, standing, and physical workload have a substantial impact on spontaneous abortion [33], preterm delivery, low birthweight, small for gestational age (SGA), preeclampsia, and gestational hypertension [32]. Lifting items weighing over 20 kg at work more than ten times a day poses a greater risk of premature birth for working pregnant women [34]. Even if it lowers their productivity and daily wages/piece rates because of missed work targets, heavy lifting and greater heat exposure have a detrimental effect on the physical health of female brick workers [35]. During pregnancy, dehydration caused by physical exercise or occupational heat stress may cause a minor increase in uterine contractions [36]. Heat stress causes the body to lose water, which causes the uterus to contract by releasing antidiuretic hormone and oxytocin [37].

### Heat stress and pregnancy outcome

Heat is teratogenic at all stages of development, which has substantial implications for maternal health, as it affects placental blood flow and increases the risk of hypertensive crises and stillbirth by increasing vasoactive substance production, blood viscosity, and endothelial cell activity [38]. Internal and external sources of heat, such as hot tubs, saunas, and electric blankets, can cause fevers ranging from 37.8°C to over 38.9°C that can even reach 43°C [39]. Elevated ambient temperature has been shown to have a negative influence on the reproductive function [40–41] and may result in a longer gestation time [42] during the early and late stages of embryonic development. Long-term exposure to heat beyond the threshold levels has been linked to heat strain symptoms in workers [43], and pregnant women and those with co-morbidities are particularly sensitive to heat-related illnesses (HRIs) [44]. In addition, the manner in which women deal with gynaecological issues under heat stress may have a different effect on pregnancy compared to normal conditions [45]. When working in extreme heat, pregnant women are more susceptible to dizziness, fainting, nausea and vomiting, febrile chills, migraines, and sunstroke, as well as aggravation of pre-existing low or high blood pressure disorders. Increased sensitivity to fainting, which is a serious concern during the first trimester [46], may be caused by changes in hormone levels, impaired adaptive ability, maternal overheating, and the growing circular demand of the fetus on the mother [46]. Preeclampsia, gestational hypertension, and poor newborn outcomes have all been associated with high ambient temperatures during pregnancy [47]. Warmth increases the risk of preeclampsia in the third trimester by raising metabolic load and modifying maternal physiology, limiting the fetus’ ability to cope with the expanding fetus's physiological needs [48]. During the early and late phases of embryonic development, elevated ambient temperature has been found to have a deleterious impact on reproductive function [40] and may result in a longer gestation time [42]. In 2005, Motherisk published a systematic review and meta-analysis on maternal hyperthermia during the first trimester and the risk of Neural Tube Defects (NTDs) in humans [49].

Heat stress during the first months of pregnancy increases the likelihood of congenital deformities, especially of the central nervous system, spontaneous abortions, gestational age, or mental health anomaly, or stillbirth compared to exposures in the second or third trimesters [50,51,52,53]. Although certain studies found no associations between heat and preeclampsia, delayed labour, or antepartum or postpartum haemorrhage [54–55], pregnant women experienced more Emergency Room (ER) visits during heat waves due to an increase in cardiovascular events such as stroke and myocardial infarction [56–57]. Additionally, stress and sleep problems have also been linked to high temperatures in pregnant women [58–59]. DNA cleavage, chromatin clustering, disrupted mitosis, cytoskeleton modification, and altered circulation increase teratogenicity in pregnant women exposed to high temperatures [17]. Abortions, stillbirths, preterm deliveries, abnormalities, and intrauterine growth restriction are only a few of the prevalent obstetric disorders known as APOs that are much more common in developing nations [21]. Working women in low and middle-income nations, particularly those working outside in tropical countries, face significant levels of physical effort and heat exposure [46]. Women continue to work until childbirth for a variety of reasons, despite the fact that they are not allowed to work for more than two hours beyond their permissible working hours in an unduly hot atmosphere [32].

### Heat stress and birth outcomes

Environmental factors are associated not only with pregnancy-related outcomes but also with birth outcomes such as congenital cataract, low birthweight, and early delivery [30]. Pregnant women are not only impacted by heat, but their newborns are also at risk of developing heat-related diseases such as rashes [60]. Strand et al. examined seasonal patterns in birth outcomes as a function of ambient temperature and found significant associations with low birthweight, preterm birth, and stillbirth [61] independent of maternal ethnicity or age, and younger women are at a significantly increased risk for adverse outcomes [38, 61,62,63]. Extreme heat events worsen the incidence of congenital cardiac defects, as predicted by maximum temperatures trends [64]. Intrauterine growth retardation can occur at multiple stages of gestation, with mid- to late-stage gestational temperatures having the greatest impact [65]. The Aberdeen study indicated that mothers exposed to high ambient temperatures during the first trimester of pregnancy gave birth to infants with LBW [66]. Birth abnormalities occur when a woman is exposed to heat for the bulk of her gestational weeks [8,9,10,11, 67–68], including preterm birth, low birth weight, and infant death [16, 69,70,71,72,73,74,75]. Heat-induced dehydration can lead to overheating, changes in the structure of the fetal heart, and neural tube abnormalities (48), which can lead to disorders such as spina bifida, which has been linked to a high fever or hot tub use, especially during the first trimester (American Association News). The number of days fetuses were exposed to maximum daily temperatures of 30 °C was associated with an increased frequency of congenital heart defects in the newborn population [76], including non-critical defects such as an atrial septal defect [8]. Children delivered in heat-affected regions are more likely to suffer from at least one of a group of abnormal delivery disorders (including fetal discomfort, assisted ventilation for more than 30 minutes, and meconium aspiration syndrome) [77]. In LMICs, women from lower socioeconomic groups were more likely to give birth prematurely, with rates of 10.4% in Asia and 12.0% in Sub-Saharan Africa [78]. These young women are compelled to take low-paying jobs outside the home in order to sustain their families [79].

### Heat stress and pregnancy outcome among working pregnant women

Pregnant women who work are more susceptible to heat stroke and exhaustion because they must support the additional weight of the fetus while cooling their own and the fetus's bodies. Working outdoors during pregnancy increases the risk of heat-related diseases, which might affect the unborn child due to elevated body temperature [80]. Heat exacerbates the hazards of outdoor activities during pregnancy, including brickmaking, agriculture, and salt pans. APOs, including spontaneous abortions, are associated with hazardous chemicals and physical agents (high heat and strenuous labour), as well as ergonomic risks [81]. This review will enable researchers to develop individual and community-level strategies to protect pregnant women from heat stress, such as public health interventions and protective labour policies, by enhancing their understanding of the plight of pregnant women who are frequently exposed to heat stress, which can then be scaled up to other similar settings elsewhere.

#### Recommendations

In order to educate and raise awareness in India's vulnerable population, particularly in rural areas, community health specialists are required. To minimise heat stress, antenatal activities should be conducted in a temperature-neutral setting (i.e., facilities with air conditioning), and prolonged exposure to high temperatures should be avoided (American Academy of Pediatrics). Ergonomic treatments such as rearranging the work-rest cycle, drinking plenty of water to replenish water lost through perspiration, wearing PPE to protect them from radiant heat, and working at dawn or after sunset with adequate lighting, may also assist pregnant women working outdoors in reducing heat stress [35, 82]. Joint efforts at different stakeholder levels, such as in individual families, communities, local and regional governments, and the news media, can aid in avoiding the adverse effects of heat impacts.

## Conclusion

Pregnancy is supposed to be a happy time for women; however, APOs such as abortions, premature births, and low birthweight can make it stressful for some. This review's major findings highlight the need for greater epidemiological research to prevent occupational heat-related diseases, with an emphasis on vulnerable working pregnant women. Occupational health is a topic that is gaining a lot of attention around the world, and rising temperatures will have a big influence on the workforce, especially pregnant women in developing countries. Heat exposure and poor maternal and newborn outcomes have been related to APOs, which remain a public health concern in both developing and developed countries. Creating protective and gender-sensitive regulatory concepts and strong policies will simplify labour regulations, which may minimize maternal mortality and morbidity among the millions of pregnant working women.
